# Genome Sequencing Reveals a Large and Diverse Repertoire of Antimicrobial Peptides

**DOI:** 10.3389/fmicb.2018.02012

**Published:** 2018-08-27

**Authors:** Reza Rezaei Javan, Andries J. van Tonder, James P. King, Caroline L. Harrold, Angela B. Brueggemann

**Affiliations:** ^1^Nuffield Department of Medicine, University of Oxford, Oxford, United Kingdom; ^2^Department of Medicine, Imperial College London, London, United Kingdom

**Keywords:** bacteriocins, pneumococcus, genomics, antimicrobials, population biology

## Abstract

Competition among bacterial members of the same ecological niche is mediated by bacteriocins: antimicrobial peptides produced by bacterial species to kill other bacteria. Bacteriocins are also promising candidates for novel antimicrobials. *Streptococcus pneumoniae* (the “pneumococcus”) is a leading cause of morbidity and mortality worldwide and a frequent colonizer of the human nasopharynx. Here, 14 newly discovered bacteriocin gene clusters were identified among >6,200 pneumococcal genomes. The molecular epidemiology of the bacteriocin clusters was investigated using a large global and historical pneumococcal dataset dating from 1916. These analyses revealed extraordinary bacteriocin diversity among pneumococci and the majority of bacteriocin clusters were also found in other streptococcal species. Genomic hotspots for the integration of different bacteriocin gene clusters were discovered. Experimentally, bacteriocin genes were transcriptionally active when the pneumococcus was under stress and when two strains were co-cultured in broth. These findings reveal much more diversity among bacterial defense mechanisms than previously appreciated, which fundamentally broaden our understanding of bacteriocins relative to intraspecies and interspecies nasopharyngeal competition and bacterial population structure.

## Introduction

Competition among bacterial members of the nasopharyngeal microbiome is mediated at least in part by bacteriocins, which are ribosomally synthesized antimicrobial peptides produced by bacterial species to inhibit the growth of other closely related bacteria. The producer strain also encodes an immunity protein to protect itself from its own bacteriocin (Dawid et al., 2006; Czaplewski et al., 2016). Bacteriocin production has been associated with more efficient colonization of a host by the producer strain, owing to the ability of these peptides to remove competitors (Dawid et al., 2006; Shak et al., 2013). Their ability to kill bacteria makes bacteriocins attractive potential candidates for the development of new antimicrobials. Several bacteriocins (e.g., nisin and pediocin PA-1) have already been commercialized and are widely used as food preservatives (Czaplewski et al., 2016).

From a genetic perspective, bacteriocins are found in gene clusters, whereby the genes involved in bacteriocin production, immunity and transport (exporting and processing the bacteriocin peptide) are situated adjacent to each other in the bacterial genome. The *blp* (bacteriocin-like peptides) cluster is the best-characterized bacteriocin among pneumococci (Reichmann and Hakenbeck, 2000; Dawid et al., 2006; Shak et al., 2013; Bogaardt et al., 2015; Czaplewski et al., 2016). Previous work by our group showed that the *blp* cluster is ubiquitous among pneumococci recovered from the early 1900s onward and is highly diverse. We also identified a novel bacteriocin cluster that we named pneumocyclicin (Bogaardt et al., 2015). Five additional bacteriocins have been reported among pneumococci (Guiral et al., 2005; Begley et al., 2009; Hoover et al., 2015; Maricic et al., 2016; Kadam et al., 2017), however, their prevalence, genetic composition and molecular epidemiology in the context of the pneumococcal population are unknown.

Pneumococci are a leading cause of severe infections such as pneumonia, bacteraemia, and meningitis, and are among the most common causes of otitis media, sinusitis, and conjunctivitis. All age groups are susceptible to pneumococcal infection, but young children, the elderly, and immunocompromised persons are most at risk (Bogaert et al., 2004). Despite the use of antimicrobials and pneumococcal conjugate vaccines, the pneumococcus remains a major global health problem, causing approximately 14.5 million cases of serious disease and 826,000 deaths annually in children <5 years of age (O’Brien et al., 2009). Antimicrobial-resistant pneumococci have been a serious and increasing concern for several decades (Klugman, 1990; Doern et al., 2001; Tadesse et al., 2017). The pneumococcus is now considered to be a “priority pathogen” – defined as antimicrobial-resistant bacteria that pose the greatest threat to global health–by the World Health Organization, 2017.

The pneumococcus is normally an asymptomatic colonizer of the nasopharynx in healthy young children; however, colonization is also the initial stage of the disease process. The polysaccharide capsule is the main virulence factor of the pneumococcus: nearly 100 distinct capsular antigenic types (serotypes) have been described and certain serotypes are predominantly associated with disease whilst others are largely associated with nasopharyngeal colonization (Brueggemann et al., 2003). Pneumococci frequently co-colonize with other pneumococci and non-pneumococcal bacterial species, and intraspecies and interspecies competition can influence colonization dynamics, strain prevalence, serotype distributions and consequently, the potential for disease progression (Shak et al., 2013).

The abundance of whole genome sequence data expedites the detection and genetic analysis of bacteriocin clusters. Here, we report 14 newly discovered pneumococcal bacteriocin clusters and describe the molecular epidemiology of all the currently known bacteriocins within a collection of pneumococci isolated over the past 90 years. We also found that identical or highly similar versions of the pneumococcal bacteriocin gene clusters could be found in other unrelated streptococcal species. We provide transcriptomic evidence that multiple bacteriocin clusters were induced in response to external stress and in response to competition for space and nutrients in broth co-culture.

## Materials and Methods

### Genome Mining for Bacteriocin Clusters

In total, 6,244 assembled pneumococcal genomes from studies previously published by us and others (Croucher et al., 2011, 2013a,b; Chewapreecha et al., 2014; van Tonder et al., 2014, 2015; Gladstone et al., 2015; Brueggemann et al., 2017) as listed in the **Supplementary Table S1** were screened for the presence of bacteriocin clusters using a variety of bioinformatic tools and databases, including antiSMASH (Weber et al., 2015) (to identify putative gene clusters that encode microbial secondary metabolites), BACTIBASE (Hammami et al., 2010) and BAGEL (van Heel et al., 2013) (to screen our genome sequences for homology to known bacteriocin genes from a diverse range of bacterial species) and InterProScan (Jones et al., 2014) (to assess the putative function of encoded proteins and identify protein domains and key sites). An in-house pipeline was developed to automate part of this process. Predicted gene clusters from each of the database outputs were examined manually and further scrutinized using extensive BLAST searches. No single program or sequence alignment was sufficient to definitively identify every bacteriocin cluster in its entirety, but rather a combination of tools was used to be confident of the identification of each bacteriocin gene cluster.

### Analyses of the Putative Bacteriocin Genes

Putative bacteriocin genes were annotated using homology to other known bacteriocin genes (**Supplementary Figure S1**) in all available databases mentioned above, as well as structure-based searches. Protein domains were examined using the Conserved Domain search feature at NCBI (Marchler-Bauer et al., 2010). Genes of interest were screened against the STRING database (Szklarczyk et al., 2015) to search for any previously reported relationship to other genes. Multiple sequence alignments of the genes in streptococcin clusters were performed in Geneious version 9.1 (Biomatters Ltd.) using the ClustalW algorithm (Larkin et al., 2007) with default parameters (Gap open cost = 15, Gap extend cost = 6.66). The multiple sequence alignment output was used within the Geneious environment to calculate percentage identity matrices. The figures of the coding regions of the bacteriocin clusters and their flanking genes were generated in Geneious and edited using Inkscape^1^.

### Classification and Nomenclature of Bacteriocin Clusters

Putative bacteriocin clusters were classified based on their predicted biosynthetic machinery and structural features (Arnison et al., 2013) and their corresponding genes were designated per the standards of nomenclature for bacteriocins (**Supplementary Table S2**). BLAST searches in the NCBI database revealed that the majority of these clusters are also present in other closely related streptococci (**Supplementary Table S3**); therefore, the clusters were named using the prefix “strepto-” followed by an abbreviation of their bacteriocin class: “streptococcins” for those that were lactococcin 972-like, “streptolancidins” for lanthipeptides, “streptocyclicins” for the head-to-tail cyclized peptides, “streptosactins” for the sactipeptides, and “streptolassins” for the lassopeptide group of bacteriocins. When more than one bacteriocin cluster from the same class was present, they were lettered alphabetically by the order of their discovery in our analyses.

### Molecular Epidemiology of the Bacteriocin Clusters

We compiled a global and historical dataset (*n* = 571) by selecting a diverse set of pneumococcal genomes isolated between 1916 and 2009 from people of all ages residing in 39 different countries. Pneumococci from both carriage and disease, 88 different serotypes and 99 different clonal complexes were represented in this dataset (**Supplementary Table S4**). Genomes and their associated metadata were stored in a BIGSdb database (Jolley and Maiden, 2010). The BIGSdb database platform was used to generate a presence/absence matrix of all the known bacteriocin genes in all 571 genomes. (Note that there were two small frameshifted gene remnants of both streptolancidins C and E present in some genomes, but these were not analyzed further in this study.) Using this matrix (79,940 genes) as an input, an in-house python script was developed (available upon request) to calculate the prevalence, molecular epidemiology, and co-occurrence patterns of all the bacteriocin clusters in the study dataset.

### Construction of the Core Genome Phylogenetic Tree

All genomes in the study dataset were annotated using the Prokka prokaryotic annotation pipeline (Seemann, 2014). The annotation files were input into Roary (Page et al., 2015) and clustered using a sequence identity threshold of 90%. Genes present in every genome were selected using a core genome threshold of 100% and were aligned using Roary. FastTreeMP (Price et al., 2010) was used to construct the phylogenetic tree using generalized time-reversible model (parameters: FastTreeMP -nt -gtr). ClonalFrameML (Didelot and Wilson, 2015) was then applied to reconstruct the phylogenetic tree adjusted for recombination. The tree was annotated using iTOL (Letunic and Bork, 2016) and Inkscape.

### Investigation of Bacteriocin Cluster Insertion Sites

Bacteriocin cluster sequences were used as queries to BLAST against genomes in the study dataset using the custom BLAST implemented in Geneious. The matching region plus additional flanking regions were visualized using the query-centric alignment feature within the Geneious environment. Regions of DNA with different bacteriocin clusters but identical flanking genes among different isolates were identified and further investigated using the Artemis Comparison Tool (ACT) (Carver et al., 2005). Linear comparison figures were generated using Geneious, ACT, and Inkscape.

### RNA Sequencing Analyses

In the first experiment, total bacterial RNA sequencing was performed on RNA extracted from pneumococcal strain 2/2 grown at a higher incubation temperature than normal (40°C vs. 37°C) to induce a bacterial stress response (Kurioka et al., 2017). Pneumococci were grown in brain–heart infusion broth for 6 h and RNA extractions were performed on samples from five time points (2, 3, 4, 5, and 6 h of incubation) using the Promega Maxwell^®^ 16 Instrument and LEV simplyRNA Cells purification kit, following the manufacturer’s protocol. Extracted RNA samples were sent to the Oxford Genomics Centre for sequencing on the Illumina platform (NCBI GEO accession number GSE103778). The sequenced forward and reverse reads were paired and mapped onto the annotated pneumococcal strain 2/2 genome using Bowtie2 (Langmead and Salzberg, 2012) with the highest sensitivity option. Differential gene expression was assessed in Geneious using the DESeq (Anders and Huber, 2010) method. Genes with an adjusted *P*-value <0.05 were deemed to be differentially expressed.

In a second experiment, pneumococcal reference strains PMEN-3 (Spain^9*V*^-3) and PMEN-6 (Hungary^19*A*^-6) were grown together in brain–heart infusion broth for 6 h. The controls were prepared by growing each strain individually in brain–heart infusion broth for 6 h. Total bacterial RNA sequencing was performed on RNA extracted from broth cultures at 2, 3, 4, 5, and 6 h after incubation using the procedures described above (NCBI GEO accession number GSE110750).

A pseudo-reference genome was constructed using Bowtie2, Velvet (Zerbino and Birney, 2008), and MeDuSa (Bosi et al., 2015) to sort genes into three categories: those unique to PMEN-3, those unique to PMEN-6, and those shared between the two (**Supplementary Figure S2**). The RNA sequencing reads for each individual control strain at all time points were pooled *in silico*. Data from all time points were combined to minimize variability caused by different growth rates of strains. Data from all time points sampled in the broth culture of PMEN-3 + PMEN-6 were also combined *in silico* and mapped to the pseudo-reference genome using Bowtie2 with the highest sensitivity option. Differential expression analyses were performed using the DESeq method by comparing sequence reads generated when strains were co-cultured to those from when strains were grown individually (**Supplementary Figure S2**).

Theoretically, the control contained double the amount of reads in comparison to the *in vivo* competition experiment due to being compiled *in silico* from two sets of samples. While this meant that the downregulation of genes could not reliably be assessed, one could have confidence that upregulated genes were differentially expressed (since expression levels must exceed that of the combined controls). However, this approach can provide only relative and not absolute values, and true fold-change ratios for the upregulated genes are most likely underestimated using this method.

## Results

### Genome Mining Triples the Number of Known Bacteriocins Among Pneumococci

Our investigation of a large and diverse dataset of 571 historical and modern pneumococcal genomes resulted in the identification of 14 newly discovered bacteriocin clusters, increasing the number of known bacteriocins in this species to 21 (**Table 1**). We identified several clusters similar to lactococcin 972, sactipeptide and lassopeptide bacteriocins (Martińez et al., 1999; Arnison et al., 2013; Letzel et al., 2014), which were hitherto not known to be harbored by the pneumococcus (**Table 1** and **Supplementary Table S2**). We subsequently expanded our search for bacteriocins to a much larger dataset of 5,673 published pneumococcal genomes, but no additional bacteriocins were found; thus, the detailed description of the bacteriocins here is restricted to those identified in the dataset of 571 genomes.

**Table 1:** Bacteriocin clusters identified among a dataset of 571 pneumococci recovered since 1916 from patients of all ages residing in 39 different countries.

Bacteriocin	Genome(s)			Year(s) of isolation	Countries (*n*)	CCs^a^ (*n*)	Serotypes (*n*)
					
	Complete	Partial	Total				
Streptococcin A	404 (70.8%)	1 (0.2%)	405 (70.9%)	1916–2009	36	80	76
Streptococcin B	414 (72.5%)	157 (27.5%)	571 (100%)	1916–2009	39	99	88
Streptococcin C	571 (100%)	0 (0.0%)	571 (100%)	1916–2009	39	99	88
Streptococcin D	6 (1.1%)	0 (0.0%)	6 (1.1%)	1968–2005	5	3	4
Streptococcin E	93 (16.3%)	477 (83.5%)	570 (99.8%)	1916–2009	39	98	88
Streptolancidin A^b^	1 (0.2%)	1 (0.2%)	2 (0.4%)	1972–2006	2	2	2
Streptolancidin B^c^	49 (8.6%)	44 (7.7%)	93 (16.3%)	1939–2006	16	11	10
Streptolancidin C	96 (16.8%)	0 (0.0%)	96 (16.8%)	1937–2006	13	15	12
Streptolancidin D	49 (8.6%)	0 (0.0%)	49 (8.6%)	1938–2006	13	13	11
Streptolancidin E^d^	12 (2.1%)	156 (27.3%)	168 (29.4%)	1937–2009	26	21	38
Streptolancidin F	23 (4.0%)	0 (0.0%)	23 (4.0%)	1937–2006	7	4	11
Streptolancidin G^e^	190 (33.3%)	9 (1.6%)	199 (34.9%)	1916–2009	25	38	46
Streptolancidin H	1 (0.2%)	0 (0.0%)	1 (0.2%)	2006–2008	1	0	1
Streptolancidin I	1 (0.2%)	0 (0.0%)	1 (0.2%)	2009	1	1	1
Streptolancidin J	185 (32.4%)	195 (34.2%)	380 (66.5%)	1916–2009	33	64	74
Streptolancidin K	1 (0.2%)	10 (1.8%)	11 (1.9%)	1943–2009	4	2	3
Streptocyclicin^f^	209 (36.6%)	0 (0.0%)	209 (36.6%)	1937–2009	20	46	59
Streptolassin	20 (3.5%)	0 (0.0%)	20 (3.5%)	1939–1996	8	7	10
Streptosactin	1 (0.2%)	0 (0.0%)	1 (0.2)	2009	1	1	1
cib^g^	557 (97.5%)	0 (0.0%)	557 (97.5%)	1916–2009	39	97	88
blp^h^	N/A	N/A	571 (100%)	1916–2009	39	99	88


^a^CC, clonal complex (genetic lineage). Singletons (single genotypes with no closely related variant) were excluded from the count. Synonym(s) for the previously identified bacteriocins are as follows: ^*b*^Pneumolancidin (Maricic et al., 2016) and salivaricin E (Walker et al., 2016); ^*c*^lcpAMT (Kadam et al., 2017) and ICESp23FST81 lantibiotic (Croucher et al., 2008); ^*d*^SP23-BS72 lantibiotic (Begley et al., 2009); ^*e*^Phr lantibiotic (Hoover et al., 2015); ^*f*^Pneumocyclicin (Bogaardt et al., 2015); ^*g*^cibAB (Guiral et al., 2005); ^*h*^spi and pnc (Bogaardt et al., 2015).

**FIGURE 1:**
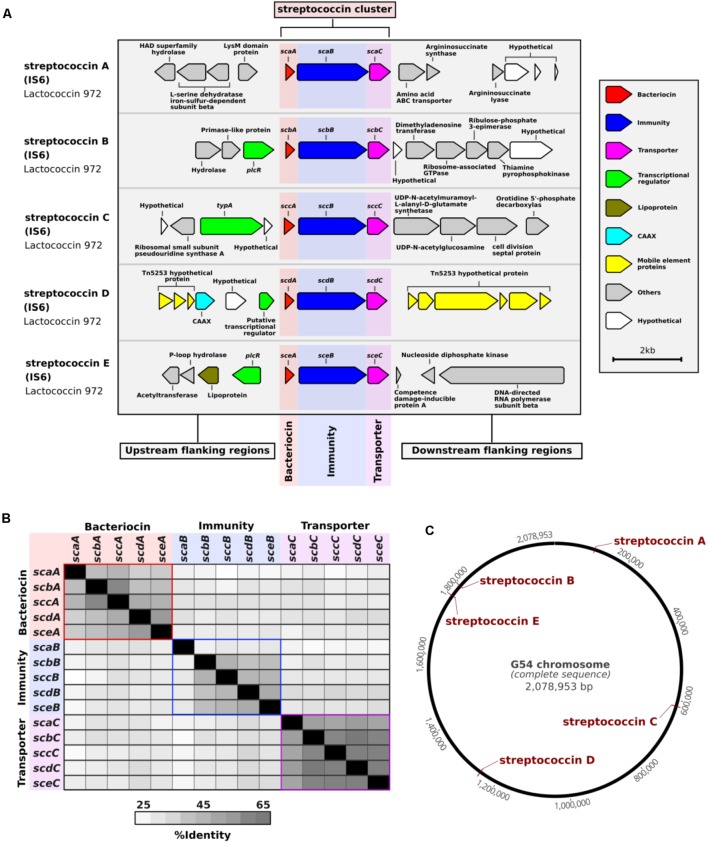
Streptococcin bacteriocins discovered among a large collection of pneumococcal genomes. **(A)** A schematic representation of each streptococcin cluster and their flanking regions is depicted. The coding regions were derived from the genome of pneumococcal strain IS6, which harbors five different complete streptococcin clusters. **(B)** A distance matrix of the nucleotide sequence identity shared between genes of different streptococcin clusters found in pneumococcal strain IS6 is shown. **(C)** Schematic of the finished genome of pneumococcal strain G54 with the locations of the five streptococcin clusters highlighted in red.

**FIGURE 2:**
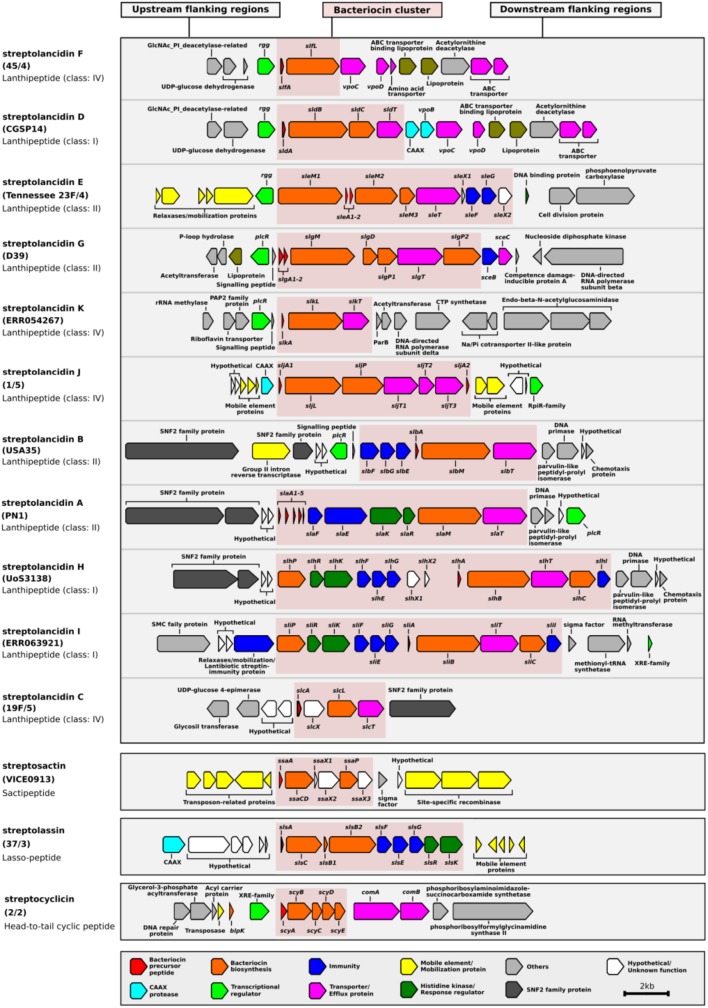
Genetic composition of streptolancidin, streptocyclicin, streptosactin, and streptolassin bacteriocins in the pneumococcal genomes. A schematic representation of 11 different streptolancidins, one streptocyclicin, one streptosactin, and one streptolassin identified among pneumococcal genomes is depicted. The names of the isolates containing each bacteriocin cluster are given in brackets and the class of each bacteriocin cluster is given underneath the names of isolates.

BLAST searches of the NCBI database of non-redundant nucleotide sequences revealed that the majority of these clusters were not exclusive to pneumococcus, but that identical or similar versions were present in other streptococci (**Supplementary Table S3**). Therefore, we used the prefix “strepto” and named each cluster according to its type of bacteriocin. Among the putative bacteriocins, five were similar to lactococcin 972 (Martińez et al., 1999) and we called these streptococcins (**Figure 1** and **Supplementary Figure S1**). Despite gene synteny among the streptococcins (**Figure 1A**), nucleotide sequence similarity was low: bacteriocin genes, 37–59%; immunity genes, 27–48%; and transporter genes, 49–63% (**Figure 1B**). Different streptococcins were found in different, but consistent, locations within the bacterial chromosome (**Figures 1A,C**). Eleven different streptolancidins, one streptosactin, one streptolassin, and one streptocyclicin [what we previously called pneumocyclicin (Bogaardt et al., 2015)] were also identified among pneumococcal genomes, as shown in **Figure 2** (Arnison et al., 2013 and **Supplementary Figure S1**). The bacteriocin clusters were commonly flanked by genes predicted to encode CAAX proteins (possibly involved in self-immunity from bacteriocins; Kjos et al., 2010), Rgg and PlcR transcriptional regulators (implicated in bacterial quorum sensing; Perez-Pascual et al., 2016), transporters, lipoproteins and mobilization proteins.

Further support for interspecies exchange of bacteriocin gene clusters was provided by the guanine (G) and cytosine (C) content of the bacteriocin clusters. The average GC-content of all pneumococcal genomes in this dataset was 39.6%, whereas the range of values for different bacteriocin groups was as follows: streptococcins, 36.3–42.4%; streptolancidins subset 1, 31.2–33.4%; streptolassin, 31.2%; streptolancidins subset 2, 28.9–29.6%; streptocyclicin, 27.0%; and streptosactin, 25.5% (**Supplementary Figure S3**). For comparison, the GC-content of other non-pneumococcal *Streptococcus* species in another study ranged between 33.2 and 44.6% (Kurioka et al., 2017 and **Supplementary Figure S3**).

### Extraordinary Bacteriocin Diversity Within a Globally Distributed Pneumococcal Dataset Dating From 1916

**FIGURE 3:**
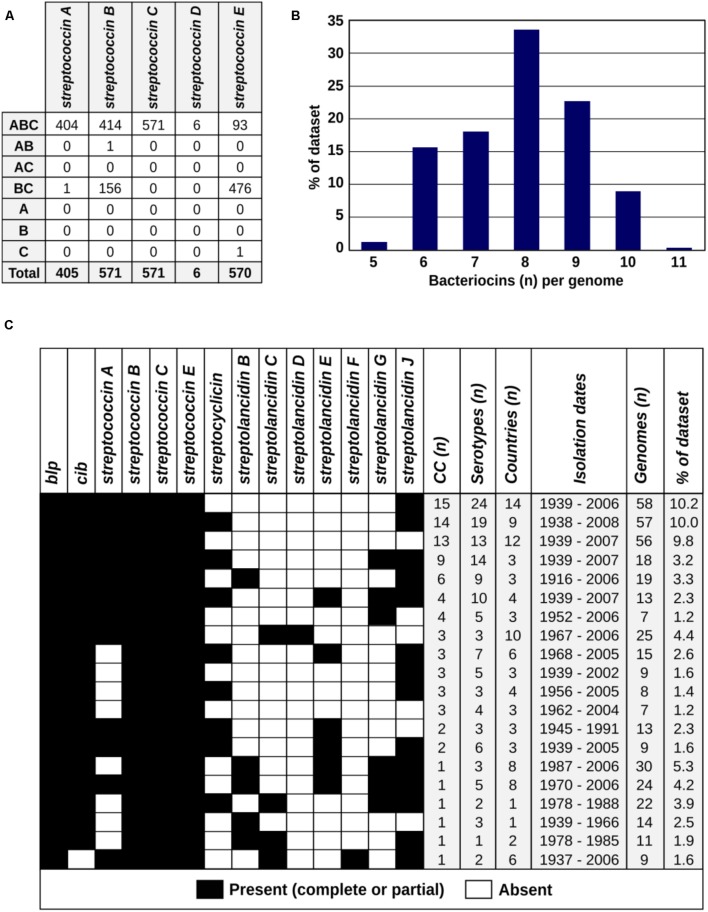
The prevalence, molecular epidemiology and co-occurrence patterns of bacteriocins within the global pneumococcal population. **(A)** The patterns of gene presence (column 1: A, bacteriocin; B, immunity; C, transporter) and frequency of those patterns (indicated by numbers) among streptococcins A–E within the study dataset are shown. **(B)** The number of bacteriocins (of any type) per genome among the 571 pneumococcal genomes is summarized. **(C)** Bacteriocin combinations found in >1 percent of the genomes in the dataset are presented as black and white boxes indicating the presence or absence, respectively, of each bacteriocin. The epidemiological characteristics of the pneumococci that possessed each combination of bacteriocins are summarized in the columns to the right of the black and white boxes.

We further assessed these bacteriocins in the context of the pneumococcal population structure. The study dataset consisted of a diverse collection of 571 pneumococci isolated between 1916 and 2009 from patients and healthy individuals of all ages residing in 39 different countries across six continents. Eighty-eight pneumococcal serotypes and 99 different clonal complexes were represented (**Table 1** and **Supplementary Table S4**). All bacteriocins detected more than once in the dataset were identified among pneumococci isolated over several decades and from a variety of different countries (**Table 1**). Some bacteriocins were present in all pneumococci, whilst others were limited to specific clonal complexes (genetic lineages). Bacteriocin clusters missing one or more genes relative to the largest clearly defined cluster in the group were defined as partial clusters. The percentages of partial and complete clusters varied between different bacteriocins, e.g., streptococcin E was present in all apart from one pneumococcal genome but as a partial cluster in the majority of genomes, while streptococcin C was found in all genomes as a complete cluster (**Table 1** and **Figure 3A**). We constructed a core genome phylogenetic tree of all isolates and labeled each genome according to the presence or absence of each bacteriocin cluster (**Supplementary Figure S4**). Overall, we found that the number of bacteriocins within each genome varied from 5 to 11 per genome and that certain combinations of bacteriocins were well represented in the dataset (**Figures 3B,C** and **Supplementary Figure S4**).

Due to their relative simplicity (containing only three genes), we chose streptococcins as models for further investigating the pattern of missing genes in partial clusters (**Figure 3A**). Scrutinizing these clusters revealed that the majority of the partial clusters lack the bacteriocin gene, while still retaining the immunity and/or the transporter genes. This could support the general idea of a “cheater” phenotype, whereby the immunity and transporter genes are conserved to protect the pneumococcus from neighboring bacteria that express the bacteriocin, but the cheater strain does not bear the cost of producing the bacteriocin (Brown et al., 2009; Son et al., 2011).

### Multiple Hotspots for the Integration of Bacteriocin Clusters in the Pneumococcal Genome

**FIGURE 4:**
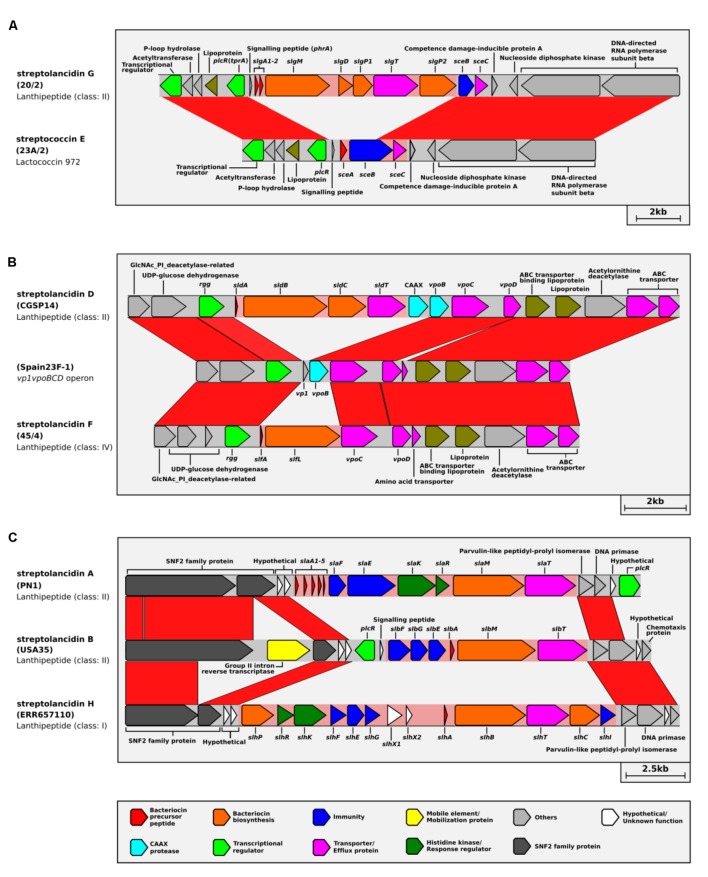
Whole-genome-based population analysis reveals evidence for bacteriocin switching. Bacteriocin cluster hotspots (BCHs) were defined as regions of DNA where different bacteriocin clusters with identical flanking genes were found among pneumococcal genomes. Linear comparisons of **(A)** BCH-1, **(B)** BCH-2, and **(C)** BCH-3 are shown. The isolate names are given in brackets. The class of each bacteriocin cluster is given underneath the isolate name.

By conducting a population genomics-based assessment of the bacteriocin cluster insertion sites, we identified three genomic regions that are putative hotspots for the integration of bacteriocin clusters in the pneumococcal chromosome. These bacteriocin cluster hotspots (BCHs) are specific locations in the genome where different bacteriocin clusters were found in different pneumococci (**Figures 4A–C**). This suggested a switching mechanism whereby different clusters can replace one another via homologous recombination. Up to three different bacteriocin clusters were found to be associated with a single BCH (**Figure 4C**). The acquisition of streptolancidin G appears to have rendered the streptococcin E partial by replacing the bacteriocin and part of its immunity gene (**Figure 4A**), which is in accordance with the fact that streptolancidin G could not be found in genomes that harbored a complete streptococcin E cluster (**Supplementary Figure S4**). Nonetheless, the remnant genes of the streptococcin E partial clusters were conserved in samples collected over many decades (**Figure 3A**).

### Bacteriocin Gene Expression in Response to Heat Stress and Strain Competition

**FIGURE 5:**
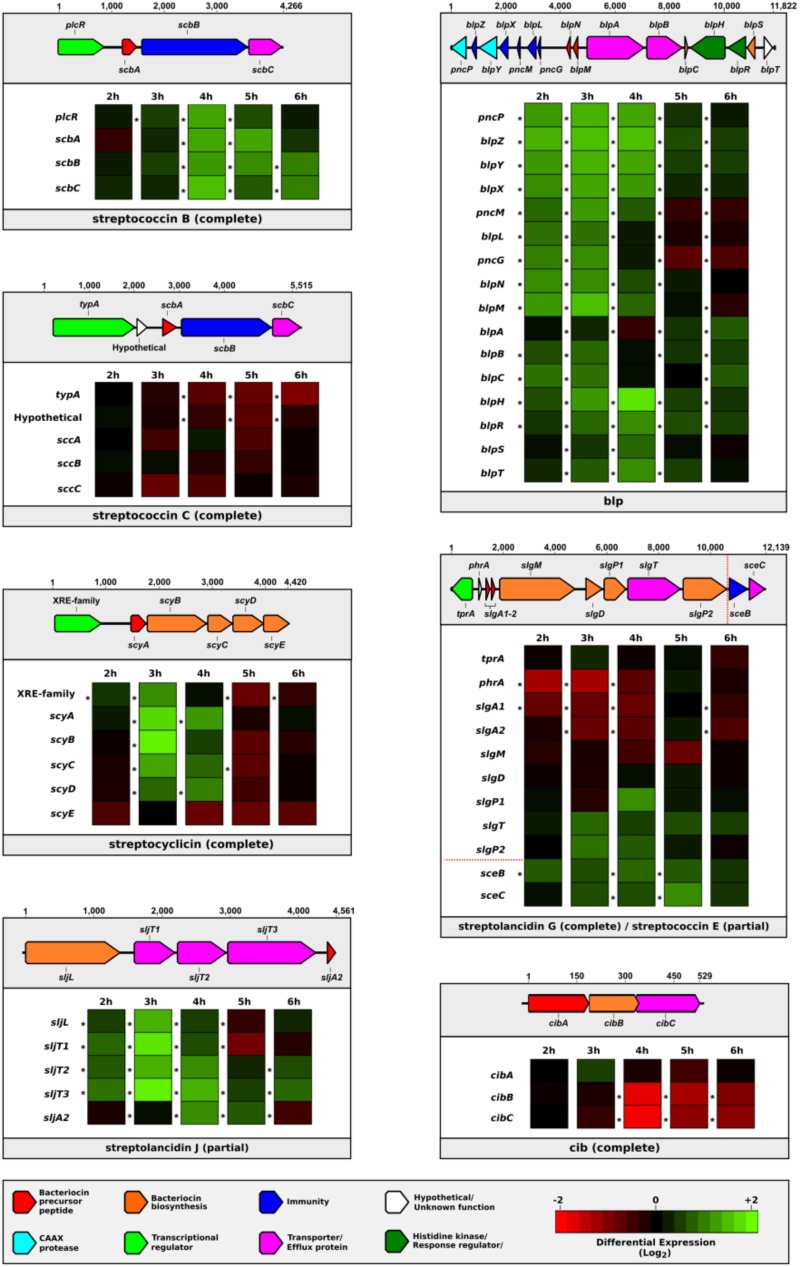
Dynamic changes in the expression of bacteriocin genes in response to bacterial stress. The differential expression levels of bacteriocin gene clusters found in pneumococcal strain 2/2 when incubated at 40°C vs. 37°C are displayed in individual boxes. A schematic representation of each bacteriocin cluster is provided above each box. Genes are represented by rows and differential expression levels at different time points are indicated in columns. An asterisk to the left of a cell indicates a statistically significant differential expression level (*p* < 0.05).

To explore whether the bacteriocin clusters were transcriptionally active, we analyzed a whole-genome RNA sequencing dataset from a broth culture of pneumococcal strain 2/2, which was incubated at a higher than normal temperature (40 vs. 37°C) to induce a bacterial stress response (NCBI GEO accession number GSE103778; Kurioka et al., 2017). Multiple genes in the bacteriocin clusters were differentially expressed compared to the control over several time points, indicating that many of these bacteriocin genes were transcribed in response to external stress (**Figure 5**).

Notably, there were two bacteriocin clusters (streptolancidin J and streptococcin E) that were missing genes and yet the remaining genes were clearly being upregulated. Moreover, the timing of gene expression varied across bacteriocin clusters, e.g., genes within a cluster were induced in a specific pattern, whilst at any particular time point during the sampling period several genes in different clusters were upregulated simultaneously. The location of and variation within the promoter regions, and the regulatory governance over which bacteriocin genes are expressed at any given point during bacterial growth remain to be determined.

A second whole-genome RNA sequencing experiment was designed to test whether bacteriocin genes were transcribed when two genetically different reference strains, PMEN-3 and PMEN-6, were cultured together in the same broth culture using standard incubation conditions. These strains were competing for space and nutrients as the incubation time progressed and cell density increased. Samples were taken for RNA sequencing at multiple time points and sequenced. Sequencing reads from all time points were combined *in silico*. The sequencing reads were mapped to a pseudo-reference genome that was constructed to include the genes unique to PMEN3 and PMEN6 plus the genes shared between both strains.

**FIGURE 6:**
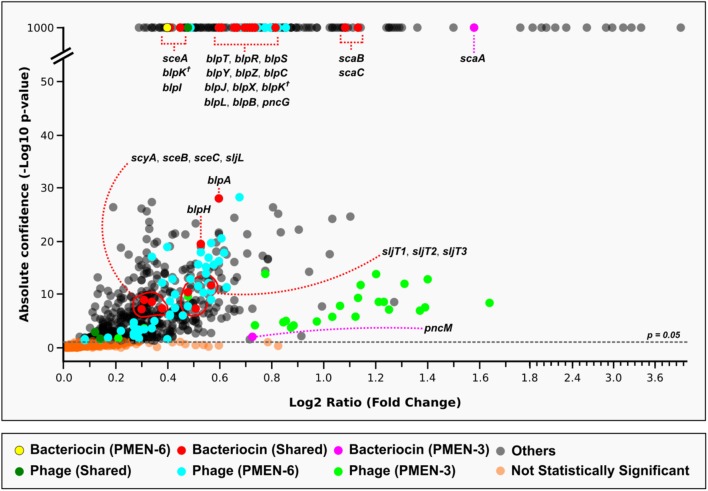
Evidence for the upregulation of bacteriocin genes when two reference strains, PMEN-3 and PMEN-6, were co-cultured in broth media. Only genes that were significantly upregulated compared to the controls (strains cultured individually) are shown in the figure. Genes of interest that were unique to each reference strain and those shared by both strains are marked in different colors. Two copies of *blpK* were found in different locations (one in the *blp* cluster as expected and one elsewhere in the genome) in both genomes and are marked here with a cross. A full list of the genes depicted here and their sequences is found in the **Supplementary Table S5**.

Pneumococcal genes that were significantly upregulated included many genes that would be expected to be expressed during growth (e.g., metabolic genes), in addition to the significant upregulation of 29 bacteriocin genes (**Figure 6** and **Supplementary Table S5**). Many of the bacteriocin genes were present in highly similar allelic versions in both PMEN-3 and PMEN-6, thus it was not possible to determine with confidence whether both versions were upregulated or whether one strain overexpressed a similar gene. However, there were three genes within the bacteriocin clusters that were unique: PMEN-3 significantly upregulated the bacteriocin gene of streptococcin A (*scaA*) and a putative immunity gene (*pncM*) from the *blp* bacteriocin cluster, and PMEN-6 significantly upregulated the bacteriocin gene of streptococcin E (*sceA*).

Interestingly, other genes that were significantly upregulated were genes associated with unique prophages present in each of the PMEN strains. We have recently demonstrated that prophage genes are ubiquitous among pneumococcal genomes, but to what extent prophages are influencing pneumococcal biology and perhaps competition between strains is not yet understood (Brueggemann et al., 2017). Overall, the data from this experiment demonstrate proof-of-principle that such methodology can be used to test for the differential expression of key bacterial genes within a competitive environment.

## Discussion

A clear understanding of the role bacteriocins play in pneumococcal biology is central to understanding microbial interactions within the ecological niche (the nasopharynx). The importance of intraspecies competition to pneumococcal ecology is reflected in the changes in prevalence of different pneumococcal serotypes and genotypes in the nasopharynx over time, and understanding competition dynamics is important in the context of understanding vaccine impact (Garcia-Rodriguez and Fresnadillo Martínez, 2002; Crook et al., 2004; Auranen et al., 2009). Pneumococcal conjugate vaccines are disruptive to the pneumococcal population structure and alter the composition of microbes competing for space and nutrients in the nasopharynx. The effects of this disruption are not yet fully understood but can lead to increased disease in human populations (Huang et al., 2005; Aguiar et al., 2010; Kaplan et al., 2010).

We revealed here that not only do pneumococci possess a substantially greater and more varied array of bacteriocins than previously recognized, the bacteriocins (often in a particular combination) are associated with specific genetic lineages. This is fundamental, as it provides the framework on which to investigate the mechanisms underpinning specific bacteriocin-pneumococcus combinations, particularly among epidemiologically successful genetic lineages, and the activity of specific bacteriocin and immunity genes. RNA sequencing clearly demonstrated that bacteriocin genes were transcriptionally active when the pneumococcus was under stress or in competition with another strain during bacterial co-culture. The *in vivo* production of bacteriocin gene products and their functional activities is under further investigation.

Interestingly, we identified several specific locations in the pneumococcal genome that harbored different bacteriocin clusters, which suggested that recombination events had occurred at these locations and resulted in a switching of bacteriocin clusters. The profound impact of recombination on the pneumococcal genome has been described for >25 years and recombination events are well documented in other locations within the pneumococcal genome, most commonly at the capsular polysaccharide locus (conferring a change of serotype) and at penicillin binding protein genes (conferring penicillin resistance if the proteins are altered) (Coffey et al., 1991; Laible et al., 1991; Maynard Smith et al., 1991; Golubchik et al., 2012; Wyres et al., 2012). Similarly, the DpnI, DpnII, and DpnIII clusters, each containing a distinct restriction modification system, can replace one another at the *dpn* locus. This is believed to be of protective value in mixed pneumococcal populations against bacteriophages, which are constant invaders of pneumococcal genomes (Johnston et al., 2013; Eutsey et al., 2015; Brueggemann et al., 2017).

We found several examples of presumed bacteriocin cluster switching events that had occurred adjacent to distinct quorum-sensing transcriptional regulators TprA and Rgg. Intriguingly, while the genes that are known to be under the control of TprA and Rgg were replaced, the quorum-sensing transcriptional regulators remained conserved. It is known that TprA controls the expression of its downstream bacteriocin genes: it induces them when pneumococcal cells are at high density in the presence of galactose and represses them when under high glucose growth conditions (Hoover et al., 2015). Galactose is plentiful in the nasopharynx, whereas glucose is scarce (although abundant in the blood), suggesting that the TprA may mediate the expression of its adjacent bacteriocin genes to aid pneumococci to compete for resources during nasopharynx colonization (Bidossi et al., 2012; Hoover et al., 2015). Likewise, the Rgg quorum-sensing transcriptional regulator has been shown to mediate the expression of its adjacent genes, and this is thought to be directed by sensing amino acid levels in the cellular community (Cuevas et al., 2017). An explanatory hypothesis might be that bacteriocin cluster switching provides a mechanism by which the existing intricate quorum-sensing signaling network required for coordinating population-level behaviors is accessible by the newly acquired bacteriocin cluster. This remains to be experimentally verified.

Our current work is significant for the broader community in that among the 21 different pneumococcal bacteriocin clusters now identified, many homologs are found in other unrelated streptococcal species. We also provide here a unified nomenclature for the pneumococcal bacteriocin clusters and their genes. Finally, the fact that any single pneumococcal genome possesses multiple bacteriocin clusters should be carefully considered when designing laboratory experiments aimed at assessing the activity of an individual bacteriocin.

Overall, these population genomic and transcriptomic analyses reveal an extraordinary complexity of bacteriocins among pneumococci and underscore the need to determine precisely how these bacteriocins drive changes within the pneumococcal population and the wider microbial community. Such findings are interesting not only for their population biology and ecology insights, but also because bacteriocins potentially have a huge impact on public health. By directly influencing changes in microbial populations, bacteriocins might indirectly be affecting the effectiveness of vaccines in the longer term: after vaccine use in human populations, the target bacterial population is significantly changed and those bacteria must now compete within the altered microbiome. If bacteriocins are essential to the microbial competitive strategy, then the composition of bacteriocins possessed by the post-vaccination bacterial community is important to understand. Moreover, there is obvious potential for the development of bacteriocins as novel antimicrobials, and at a time when the challenges of antimicrobial-resistant microbes have never been more acute these data provide many new areas of investigation.

## Data Availability

Representative examples of the newly discovered bacteriocin clusters have been deposited at GenBank under the accession numbers of MF990778–MF990796. Accession numbers for all genomes used in this study are listed in the **Supplementary Table S1**. Raw transcriptomic sequence data used in this study is available under GEO accession numbers GSE103778 and GSE110750.

## Author Contributions

RR designed the genome mining pipelines. AT, CH, and AB performed the RNA sequencing experiments. RR, AT, CH, JK, and AB analyzed the data. RR and AB wrote the manuscript. All authors reviewed and agreed on the final version of the manuscript.

## Conflict of Interest Statement

The authors declare that the research was conducted in the absence of any commercial or financial relationships that could be construed as a potential conflict of interest.

## Footnotes

1 http://inkscape.org

## Supplementary Material

FIGURE S1 Amino acid sequence alignments of bacteriocin genes identified in this study. Putative bacteriocin genes that were identified among the pneumococcal genomes in this study were aligned against similar bacteriocin genes in other bacterial species for comparison.

FIGURE S2 Methodology for analyzing the RNA sequencing data from the co-colonization experiment. **(A)** Steps involved in creating the pseudo-reference genome sequence. The name of the tool used in each step is shown in pink. **(B)** Schematic describing the combination of RNA sequence reads to assess differential expression levels.

FIGURE S3 Guanine (G) and cytosine (C) content of pneumococcal bacteriocin clusters. **(A)** Four examples of GC plots depicting the percentage GC-content of bacteriocin cluster genes (red), transcriptional regulator genes (green), and other adjacent pneumococcal genes (grey). The names of the bacteriocin and the genome in which it was found are given, with the percentage GC-content of each in brackets. Each graph depicts GC-content and adenine (A) thymine (T) content by the green and blue lines, respectively. **(B)** Average GC-content for each bacteriocin cluster type, organized by bacteriocin type or class. The lanthipeptides formed two subsets based on GC-content of <30 or >31%. **(C)** Average GC-content for 3 genomes of non-pneumococcal streptococcal species analyzed in a previous study (Kurioka et al., 2017).

FIGURE S4 Diversity of bacteriocins within a global pneumococcal dataset. A phylogenetic tree of all genomes in the study dataset is depicted and labeled according to the presence of different bacteriocins. Clusters with missing genes were defined as partial. The exceptions were the *blp* clusters: their highly diverse and complicated genetic compositions among pneumococci genomes meant that a similar classification between partial and complete clusters could not be applied (Bogaardt et al., 2015). Instead, their presence (irrespective of being partial or complete) is depicted by the green color.

TABLE S1 List of pneumococcal genomes used in this study.

TABLE S2 Classification and nomenclature of bacteriocin clusters found among pneumococci.

TABLE S3 Result of the BLAST searches of the bacteriocin clusters in the NCBI database.

TABLE S4 Descriptive data for the genomes included in the global representative dataset.

TABLE S5 RNA-seq data from the competition experiment.
