# Supplementary figures and images for: Genome Sequencing Reveals a Large and Diverse Repertoire of Antimicrobial Peptides
Source: Front Microbiol. 2018 Aug 27;9:2012. doi: 10.3389/fmicb.2018.02012 (PMC6120550; doi:10.3389/fmicb.2018.02012)

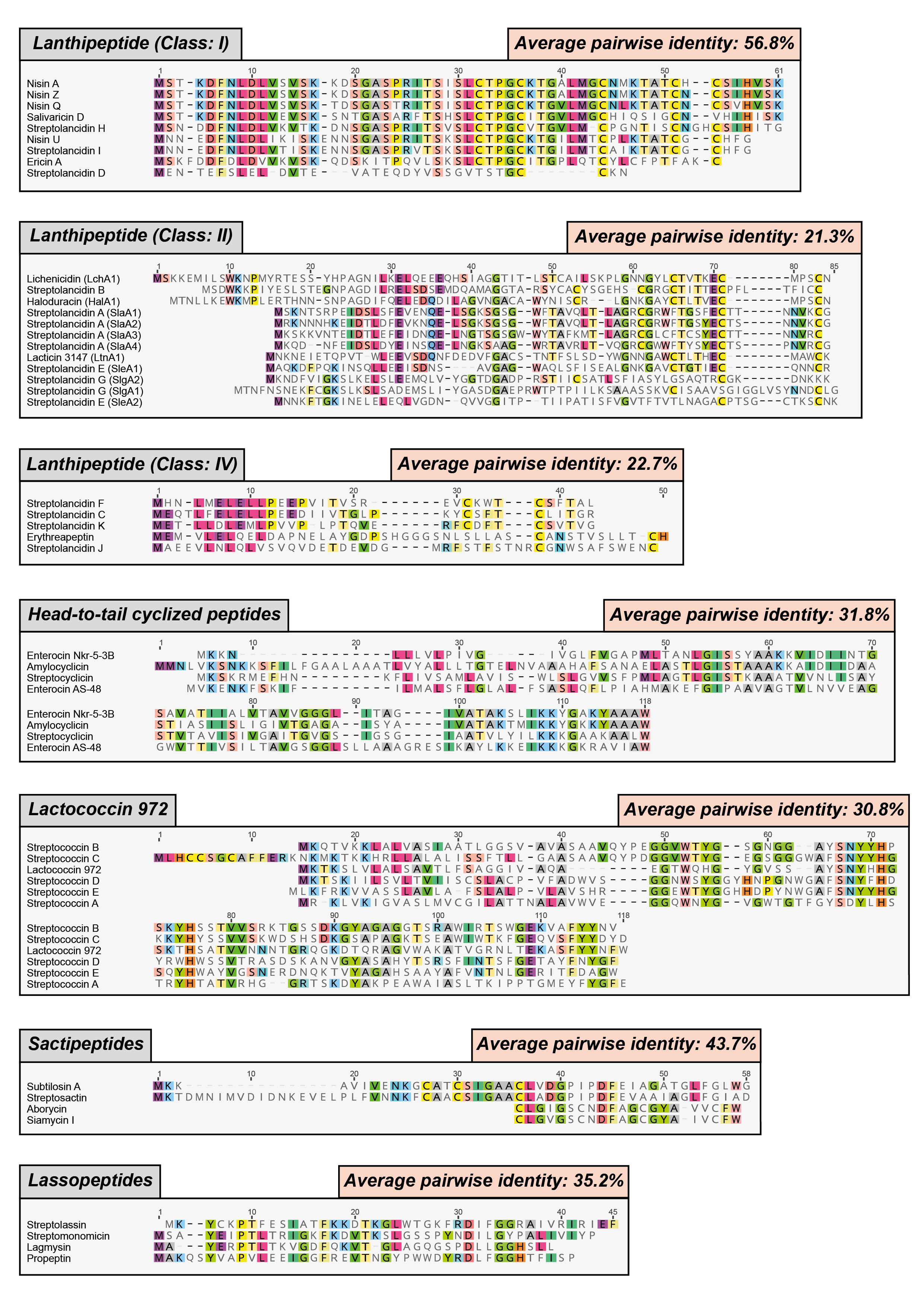

Supplement: FIGURE S1 — Amino acid sequence alignments of bacteriocin genes identified in this study. Putative bacteriocin genes that were identified among the pneumococcal genomes in this study were aligned against similar bacteriocin genes in other bacterial species for comparison. [file Image_1.TIFF]

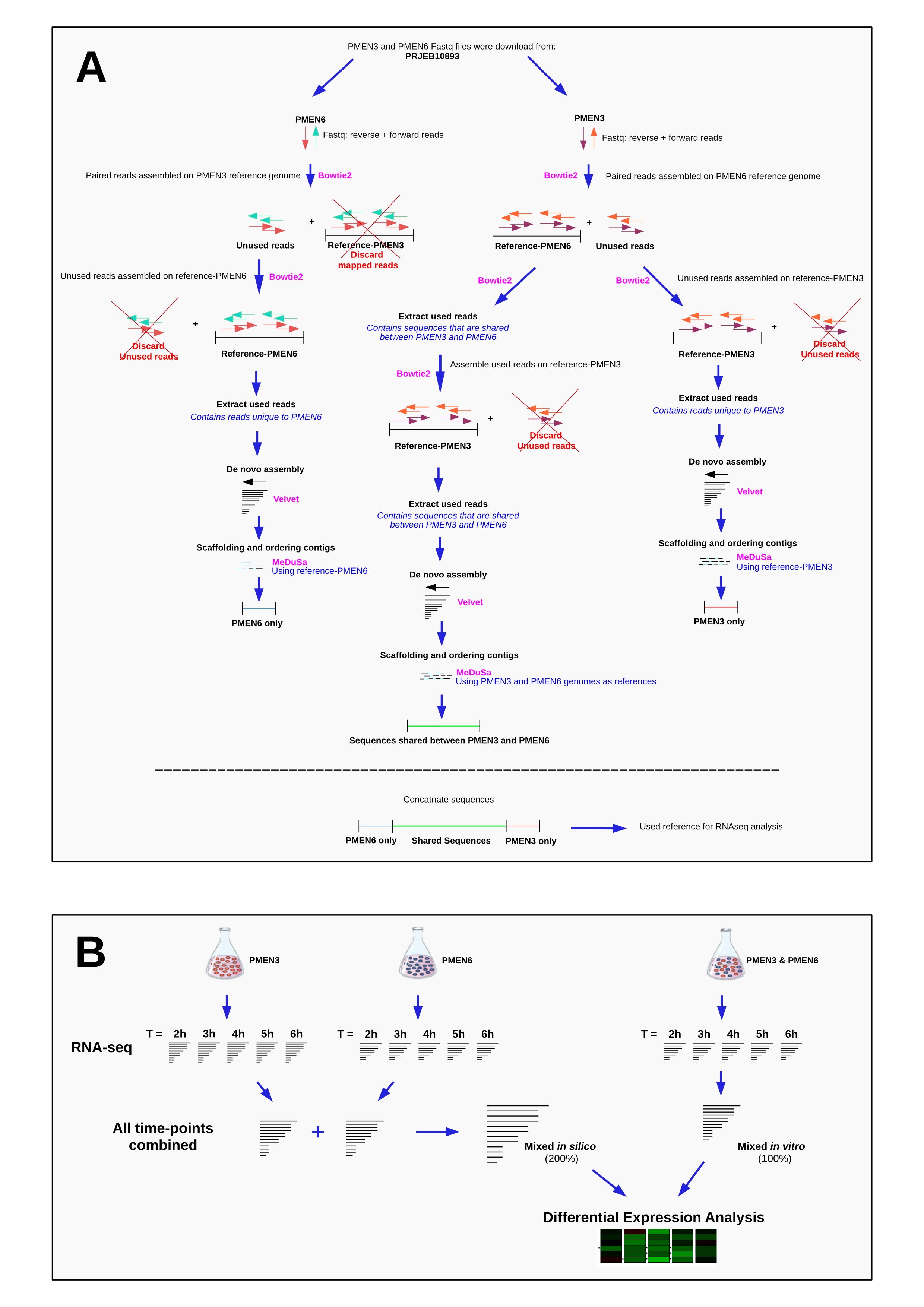

Supplement: FIGURE S2 — Methodology for analyzing the RNA sequencing data from the co-colonization experiment. (A) Steps involved in creating the pseudo-reference genome sequence. The name of the tool used in each step is shown in pink. (B) Schematic describing the combination of RNA sequence reads to assess differential expression levels. [file Image_2.TIFF]

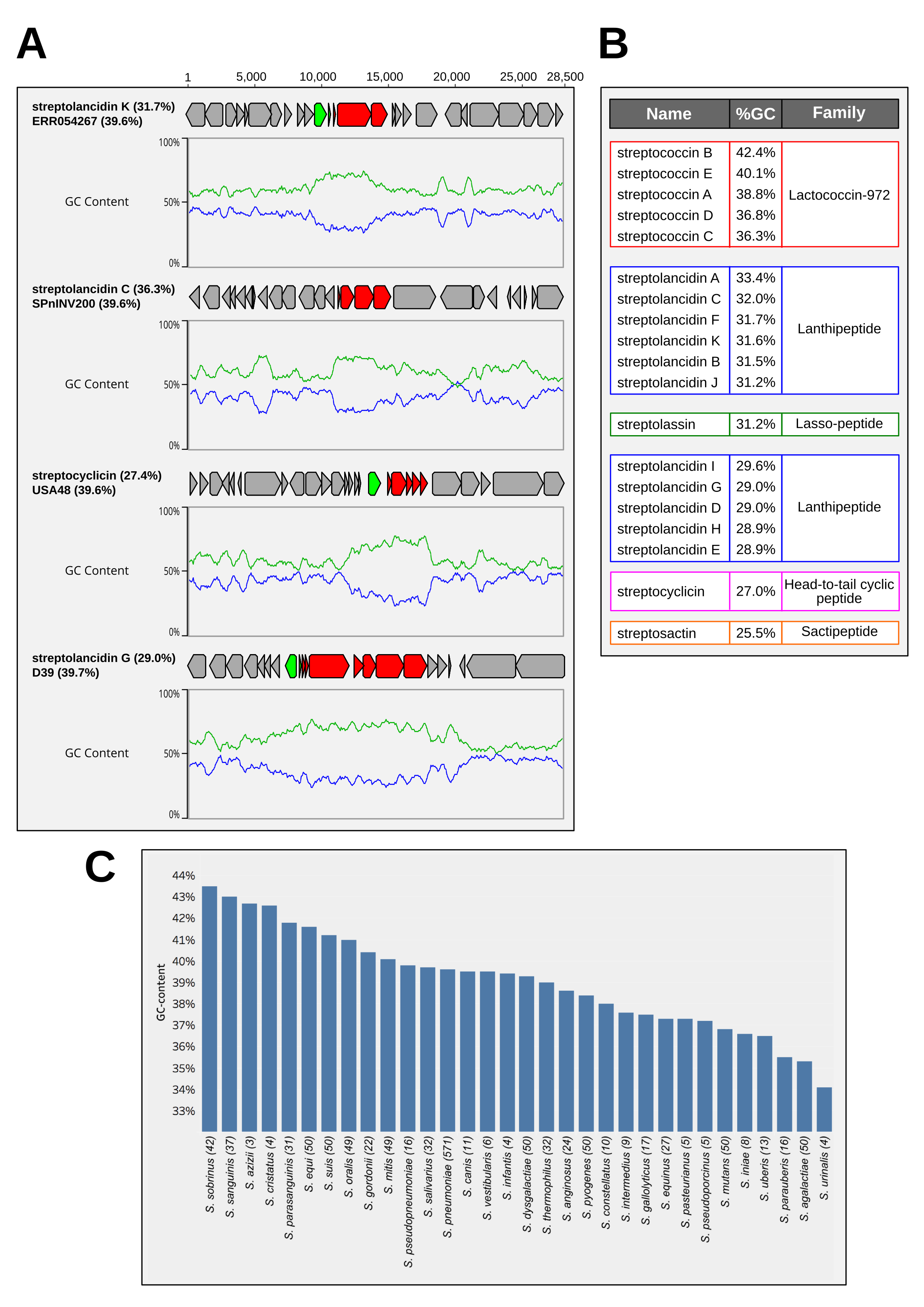

Supplement: FIGURE S3 — Guanine (G) and cytosine (C) content of pneumococcal bacteriocin clusters. (A) Four examples of GC plots depicting the percentage GC-content of bacteriocin cluster genes (red), transcriptional regulator genes (green), and other adjacent pneumococcal genes (grey). The names of the bacteriocin and the genome in which it was found are given, with the percentage GC-content of each in brackets. Each graph depicts GC-content and adenine (A) thymine (T) content by the green and blue lines, respectively. (B) Average GC-content for each bacteriocin cluster type, organized by bacteriocin type or class. The lanthipeptides formed two subsets based on GC-content of <30 or >31%. (C) Average GC-content for 3 genomes of non-pneumococcal streptococcal species analyzed in a previous study (Kurioka et al., 2017). [file Image_3.TIFF]

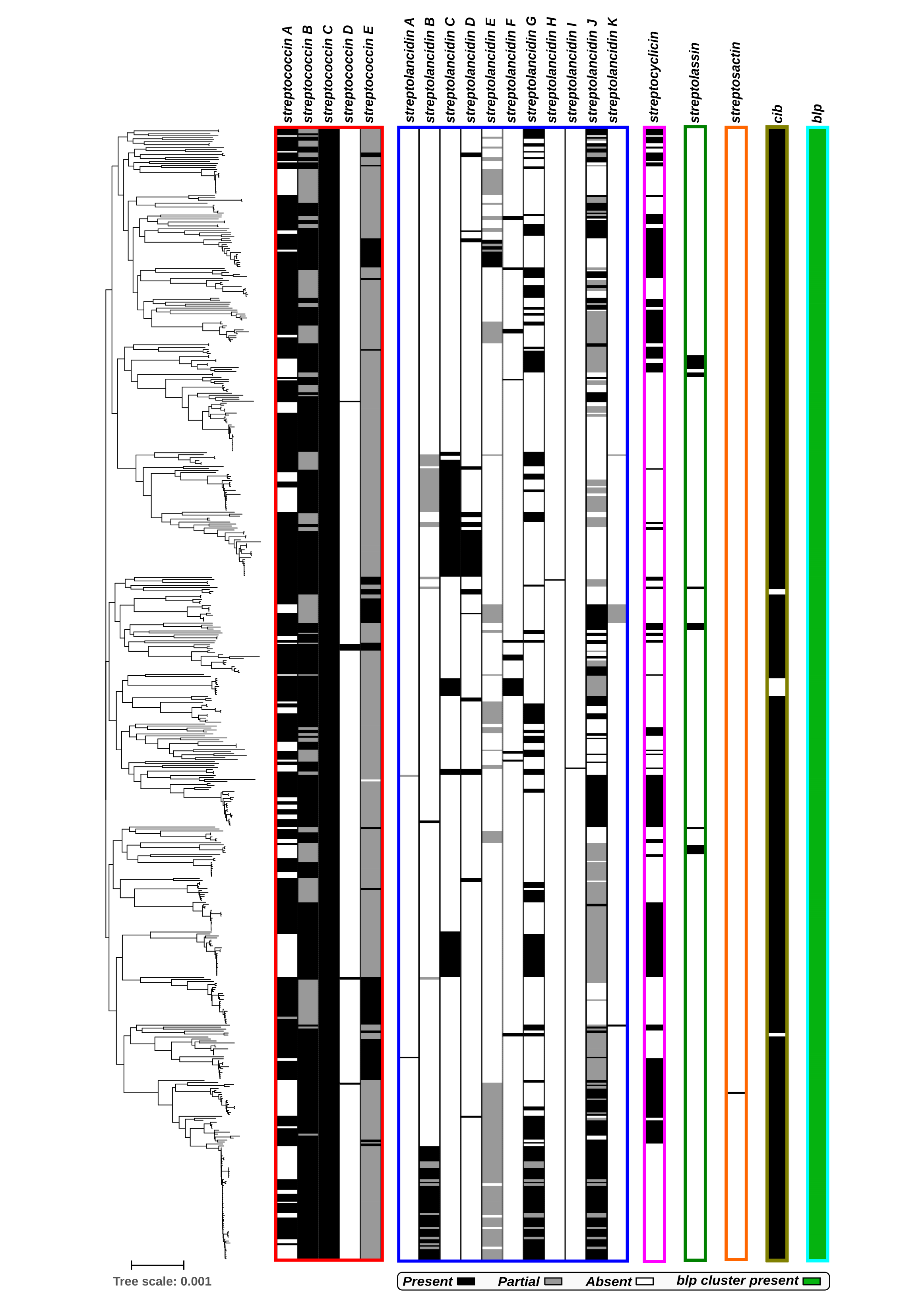

Supplement: FIGURE S4 — Diversity of bacteriocins within a global pneumococcal dataset. A phylogenetic tree of all genomes in the study dataset is depicted and labeled according to the presence of different bacteriocins. Clusters with missing genes were defined as partial. The exceptions were the blp clusters: their highly diverse and complicated genetic compositions among pneumococci genomes meant that a similar classification between partial and complete clusters could not be applied (Bogaardt et al., 2015). Instead, their presence (irrespective of being partial or complete) is depicted by the green color. [file Image_4.TIFF]
